# Analysis of the correlation between clinical and imaging features of malignant lung nodules and pathological types

**DOI:** 10.3389/fsurg.2023.1321118

**Published:** 2023-12-22

**Authors:** Liwen Zhang, Rong Wan, Jixiang Chen, Fan Xin, He Han

**Affiliations:** ^1^ Respiratory and Critical Care Department, Affiliated Hospital of Jiangsu University, Zhenjiang, Jiangsu Province, China; ^2^ Department of General Surgery, Affiliated Hospital of Jiangsu University, Zhenjiang, Jiangsu Province, China

**Keywords:** malignant pulmonary nodules, clinical features, imaging features, pathologic types, correlation analysis

## Abstract

**Introduction:**

To explore the correlation between clinical and imaging features of malignant lung nodules and pathology types.

**Methods:**

Patients with lung nodules admitted to the Affiliated Hospital of Jiangsu University from January 1, 2020 to December 31, 2020 were collected as study subjects, and all of them underwent surgical treatment and were clearly diagnosed by pathology. The correlation between clinical and imaging features and pathological types of lung cancer patients was analyzed.

**Results:**

Among them, The pathological types of malignant pulmonary nodules are correlated with age, gender, smoking history, ground glass sign, nodule size, solid to solid ratio, lobulation sign, pleural indentation sign, hair prick sign, CEA, SCCA. The imaging features of ground glass sign and nodule size are most significantly correlated with the pathological type.

**Conclusion:**

It was found that, the clinical and imaging characteristics of patients with malignant lung nodules have a certain correlation with the pathological type, and gender, age, smoking history, nodule size, nodule nature, burr sign, pleural depression sign, and tumor markers are of great value for pathological typing.

## 1. Introduction

Lung cancer is one of the malignant tumors with a high mortality rate, most of which are detected at an advanced stage, and the overall 5-year survival rate is low; early detection and intervention can reduce the mortality rate (1, 2). The overall 5-year survival rate of lung cancer is less than 15%. The 5-year survival rate of early-stage lung cancer (especially stage Ia lung cancer) after surgical resection can reach more than 90%, while the 5-year survival rate of intermediate and advanced lung cancer after surgical resection is less than 5% (3). With the wide application of low-dose spiral CT in the early detection of lung cancer, the detection rate of early-stage lung cancer, mainly characterized by lung nodules, has increased gradually (4). Relevant studies have shown that the morphology and imaging characteristics of lung nodules are closely related to their pathological types (5), Therefore, it is of great practical significance to analyze the correlation between the morphology and imaging characteristics of pulmonary nodules and the type of pathology to guide clinical diagnosis and treatment.

This study retrospectively included 202 patients with lung adenocarcinoma and squamous cell carcinoma who underwent surgical resection and pathologically confirmed diagnosis at the Department of Thoracic Surgery, Affiliated Hospital of Jiangsu University, from January 2020 to December 2020, to summarize and analyze the correlation between the clinical and imaging characteristics of the patients with malignant lung nodules and the type of pathology, so as to provide a reference for the clinical diagnosis and treatment of lung nodules.

## 2. Data and methods

### 2.1. General information

Patients with lung nodules who underwent surgical resection and pathologically confirmed diagnosis at the Department of Thoracic Surgery of Jiangsu University University Hospital from January 1, 2020 to December 31, 2020, and those with a history of intrapulmonary or extrapulmonary malignant tumors within 5 years were selected, and there were a total of 248 cases, of which 78 were male and 124 were female, with the age of 28–78 years. Among them, 202 patients diagnosed with lung adenocarcinoma (LUAD) and lung squamous cell carcinoma (LUSC) were selected for analysis.

### 2.2. Data collection

Gender, age, smoking history, tumor history, calcification, burr, lobulation, pleural pull sign, vascular cluster sign, cavity, tumor diameter and laboratory examination indexes were collected from the hospital medical record system.

### 2.3. CT scanning and image analysis methods

A 256-slice spiral CT (GE Company) was used to perform continuous spiral scanning from the lung base to the lung apex. Two deputy chief physicians, who had been engaged in diagnostic imaging for more than 10 years, counted the CT features of lung nodules such as density, size, location, margin, pleural depression sign, vascular sign, and vacuolar sign.

### 2.4. Statistical processing

SPSS 26.0 software was used for data statistics and analysis. Measurement data were expressed as mean ± standard deviation (x ± s) by Kruskal–Wallis test; count data were expressed as number of cases and percentage by *χ*2 test or Fisher's exact test; Spearman's correlation was used to analyze the correlation between the pathological types of malignant lung nodules and the clinical and imaging characteristics; *P* < 0.05 was regarded as the difference was statistically significant.

## 3. Results

### 3.1. Pathologic findings

Thirty-seven cases were diagnosed as adenocarcinoma *in situ*(AIS), 21 cases of minimally invasive adenocarcinoma(MIA, 135 cases of invasive adenocarcinoma(IAC), 9 cases of squamous cell carcinoma(SC), 2 cases of large-cell carcinoma, 1 case of small-cell carcinoma(SCLC), 1 case of adenosquamous carcinoma, 3 cases of metastatic carcinoma and 2 cases of carcinoid tumors, and the remaining 37 cases of benign lesions, including 16 cases of inflammatory lesions, 9 cases of tuberculosis, 4 cases of atypical adenomatous proliferation, 2 cases of cryptococcal lungs and 1 case of sclerosing pneumocytoma.

### 3.2. Comparison of general data of patients with malignant pulmonary nodules of different pathological types

The results are shown in the Table 1. Of the 202 patients diagnosed with lung adenocarcinoma (LUAD) and lung squamous cell carcinoma (LUSC), and the differences in age, gender distribution, and smoking history among patients with malignant lung nodules of different pathological types were statistically significant (*P* < 0 05). There was no statistically significant difference in comparing the tumor histories of patients with different pathological types of malignant lung nodules (*P* > 0.05). The differences in serum carcinoembryonic antigen (CEA) and squamous cell carcinoma antigen (SCCA) levels of patients with different pathological types of malignant lung nodules were statistically significant (*P* < 0.05). The levels of glycan antigen 199 (CA199), serum glycan antigen 125 (CA125), neuron-specific enolase (NSE), cytokeratin 19 fragment antigen 21-1 (CYFRA21-1), and gastrin-releasing peptide precursor (ProGRP) were not statistically significant in patients with different types of malignant lung nodules (*P* > 0.05).

**Table 1:** Comparison of general data of patients with malignant pulmonary nodules of different pathologic types.

General information	Pathological type				*χ2/F*	*p*
	AIS (*n* = 37)	MIA (*n* = 21)	IAC (*n* = 135)	SC (*n* = 9)		
Age/years	53.22 ± 11.96	57.81 ± 12.16	60.27 ± 9	66.22 ± 7.84	15.924	0
Sex						
Male/case (%)	7 (18.92)	6 (28.57)	56 (41.48)	9 (100)	21.724	0
Female/case (%)	30 (81.08)	15 (71.43)	79 (58.52)	0 (0)		
Smoking history			D			
Yes/case (%)	4 (10.81)	0 (0)	37 (27.41)	8 (88.89)	29.472	0
No/case (%)	33 (89.19)	21 (100)	98 (72.59)	1 (11.2)		
Tumor history						
Yes/case (%)	0 (0)	1 (4.76)	9 (6.67)	1 (11.2)	3.529	0.254
No/case (%)	37 (100)	20 (95.24)	126 (93.33)	8 (88.89)		
Tumor marker						
CEA (ng·ml^−1^)	2.04 ± 1.10	1.76 ± 0.79	2.78 ± 1.71	3.31 ± 1.69	17.468	0.001
SCCA (ng·ml^−1^)	1.36 ± 0.67	1.34 ± 0.49	1.45 ± 0.84	2.34 ± 1.01	11.732	0.008
NSE (ng·ml^−1^)	4.12 ± 1.12	4.05 ± 0.69	4.41 ± 1.85	3.92 ± 0.57	0.697	0.555
CA199 (U·ml^−1^)	7.6 ± 6.95	7.17 ± 4.27	7.91 ± 8.75	5.43 ± 3.99	0.309	0.819
CA125 (U·ml^−1^)	13.96 ± 7.40	10.72 ± 5.72	13.39 ± 11.51	11.91 ± 5.17	0.546	0.651
CYFRA21 (ng·ml^−1^)	2.81 ± 0.99	2.70 ± 0.71	3.01 ± 1.13	3.62 ± 1.07	1.925	0.127
ProGRP (ng·ml^−1^)	32.24 ± 10.45	32.95 ± 9.84	33.01 ± 12.63	26.07 ± 5.82	0.992	0.397

### 3.3. Comparison of imaging characteristics between patients with malignant pulmonary nodules of different pathological types

The results are shown in the Table 2. The differences in nodule size (diameter), pleural depression sign, lobulation, burr sign, and nature of nodules were statistically significant (*P* < 0.05). There was no statistically significant difference in the proportion of vacuolar and vascular signs among different pathologic types of malignant pulmonary nodules (*P* > 0.05).

**Table 2:** Comparison of imaging features of malignant lung nodules of different pathologic types.

Imaging features	Pathological type				*χ2/F*	*p*
	AIS (*n* = 37)	MIA (*n* = 21)	IAC (*n* = 135)	SC (*n* = 9)		
Nodule size /cm	0.96 ± 0.33	1.07 ± 0.34	1.64 ± 0.59	1.87 ± 0.3	55.742	0
Pleural depression sign						
Yes/case (%)	4 (10.81)	3 (14.29)	62 (45.93)	4 (44.44)	20.502	0
No/case (%)	33 (89.19)	18 (85.71)	73 (54.07)	5 (55.56)		
Phyllotaxy						
Yes/case (%)	1 (2.7)	0 (0)	45 (33.33)	7 (77.78)	36.965	0
No/case (%)	36 (97.3)	21 (100)	90 (66.67)	2 (22.22)		
Vacuous						
Yes/case (%)	2 (5.4)	0 (0)	24 (17.78)	0 (0)	8.208	0.028
No/case (%)	35 (94.6)	21 (100)	111 (82.22)	9 (100)		
Burr sign						
Yes/case (%)	3 (8.11)	0 (0)	52 (38.52)	3 (33.33)	26.017	0
No/case (%)	34 (91.89)	21 (100)	83 (61.48)	6 (66.67)		
Nature of the nodule						
Ground-glass sign/case (%)	34 (91.89)	19 (90.48)	49 (36.3)	0 (0)	64.24	0
Subserial signs/case (%)	0 (0)	2 (9.52)	22 (16.3)	0 (0)	9.225	0.017
Factual sign/case (%)	3 (8.11)	0 (0)	64 (47.41)	9 (100)	52.867	0
Sign of vascular conglomeration						
Yes/case (%)	6 (16.22)	4 (19.05)	29 (21.48)	3 (33.33)	1.541	0.69
No/case (%)	31(83.78)	17(80.95)	106(78.52)	6(66.67)		

### 3.4. Correlation of pathological types of malignant pulmonary nodules with gender, age, smoking history, tumor history and family history of the tumor

The results are shown in the Table 3. There was a positive correlation between patients with malignant lung nodules of different pathologic types and age, proportion of males, smoking history, and tumor history (*P* < 0.05).

**Table 3:** Correlation between the type of malignant lung nodule pathology and the patient's gender, age, smoking history, tumor history, and family history of the tumor.

Pathological type	Spearman correlation analysis	
	*r*	*P*
Age	0.268	0
Gender (percentage of male)	0.281	0
Smoking history	0.321	0
Tumor history	0.149	0.034

### 3.5. Correlation of pathological types of malignant pulmonary nodules with imaging features

The results are shown in the Table 4. Patients with malignant pulmonary nodules of different pathologic types showed a significant positive correlation with nodule size, burr sign, lobulation, pleural depression sign, and solidity (*P* < 0.05), and a significant negative correlation with ground glass (*P* < 0.05).

**Table 4:** Correlation between pathological types and imaging features of malignant lung nodules.

Pathological type	Spearman correlation analysis	
	*r*	*P*
Nodule siz/cm	0.521	0
Pleural depression sign	0.3	0
Phyllotaxy	0.389	0
Vacuous	−0.129	0.068
Burr sign	0.296	0
Ground-glass sign	−0.534	0
Subserial signs	0.136	0.054
Factual sign	0.46	0
Sign of vascular conglomeration	0.073	0.301

### 3.6. Correlation of pathological types of malignant pulmonary nodules with serum tumor marker levels

The results are shown in the Table 5. There was a significant positive correlation between the pathologic types of malignant lung nodules and serum CEA and SCCA levels (*P* < 0.05). There was no significant correlation between the pathologic types of malignant nodules and serum levels of CYFRA21, CA125, NSE, TSFG, CA153, CA199, and ProGRP (*P* > 0.05).

**Table 5:** Correlation between pathological staging of malignant lung nodules and serum tumor marker levels.

Pathological type	Spearman correlation analysis	
	*r*	*P*
CEA	0.278	0
SCCA	0.141	0.046
NSE	0.026	0.712
CA199	−0.014	0.84
CA125	−0.033	0.641
CYFRA21	0.171	0.015
ProGRP_	−0.056	0.426

## 4. Discussion

Lung cancer accounts for 18.4% of cancer-related deaths worldwide (6) and is the leading cause of death from malignant tumors. Approximately 50% of patients have reached locally advanced or distant metastases by the time of discovery, resulting in limited choice of treatment options. With the widespread use of high resolution CT (HRCT) in early lung cancer screening, the detection rate of lung nodules has increased significantly, and the use of 3D technology has improved the accuracy of differentiating benign and malignant nodules (7). But among the detected pulmonary nodules, 1%–12% were ultimately diagnosed as early cancer (8). The guidelines for the management of lung nodules issued by the American College of Radiology (ACR) state that lung nodules should be stratified and regularly followed up, but their diagnostic accuracy is insufficient and specificity is low, which may lead to over-treatment of benign nodules (9).

At the same time, conventional imaging assessment is easily influenced by human subjective factors. At present, most studies focus on the identification of benign and malignant nature of lung nodules when evaluating the timing of surgery according to the diameter of the nodule, but in terms of the timing of surgery for lung nodules, carcinoma *in situ* is not yet invasive, and in fact, it does not have the characteristic hazards of malignant tumors that invade the surrounding tissues or metastasize to other places. In terms of the surgical effect of early lung cancer in the stage of carcinoma *in situ* and microinvasive carcinoma, the 5-year survival of patients with malignant lung nodules that have not yet developed invasiveness is close to 100% after surgery, and premature surgery does not improve their survival time and quality of life after treatment (10–12). while overly aggressive surgery is considered excessive treatment for patients with pulmonary nodules to some extent (13, 14). Accurate identification of early-stage lung cancer in lung nodules and surgical treatment at the right time can significantly reduce lung cancer mortality; therefore, accurate diagnosis of lung nodules is crucial.

Studies have shown that the age-specific incidence of trachea, bronchus and lung cancer is decreasing globally in males, while the age-specific incidence of trachea, bronchus and lung cancer is increasing in females (15). The results of this study showed statistically significant differences in age, gender, and smoking history among patients with different pathologic types, with a higher proportion of SC in males than in females and a higher age of onset, which may be related to the fact that males tend to have a longer history of smoking and a longer smoking history (16). Among malignant lung nodules, the proportion of adenocarcinoma was significantly higher than that of SC, and the proportion of females in adenocarcinoma patients was significantly higher than that of males, and the incidence rate was on the rise, which suggests that the incidence of adenocarcinoma of the lungs may be related to gender (17), and its causes may be related to secondhand smoke exposure and environmental pollution (15).

The results of this study showed statistically significant differences in nodule size, proportion of pleural depression sign, lobulation, burr sign and nature of malignant lung nodules in different pathologic types, indicating that these features are valuable in identifying AIS, MIA, IAC and SC. Current studies on the aggressiveness of pulmonary nodules have shown that nodule diameter is an important risk factor for assessing aggressiveness (18). After the invasive behavior of a pulmonary nodule, the nodule diameter can be a good indicator for the different degrees of invasiveness of a pulmonary nodule and for evaluating the timing of surgery, i.e., the larger the diameter of a pulmonary nodule, the higher the risk that it will be invasive (19–23).

The results of this study showed that the difference in the comparison of the diameters of malignant lung nodules of different pathologic types was statistically significant, and the diameter of nodules in SC and IAC was significantly larger than that of AIS and MIA, which is an important indicator for predicting the degree of malignancy of the lesion, especially the change in the diameter of the lung nodules in the course of the followup, and it may be helpful in determining the nature of the lesion. Lobulation, pleural depression sign and burr sign may be related to the degree of infiltration of lung cancer (19). When there is no peripheral infiltration or the scope of infiltration is relatively small, the tumor cells grow along the alveolar wall, so they mostly appear to be nearly round. with the increase of the degree and scope of tumor infiltration, the tumor cell growth speeds up, and the contraction of internal fibrous components produces a certain tugging effect on the surrounding tissues of the tumor, which leads to the formation of an irregular shape. The present study showed that in lung adenocarcinoma, and with the further increase of the degree of infiltration pleural depression, lobulation and burr sign became more and more obvious, and was positively correlated with the degree of malignancy of the pathologic type, and the correlation was lobulation, burr sign and pleural depression sign in descending order. The proportion of pleural depression and burr sign in squamous lung cancer was comparable to that of invasive adenocarcinoma, and the proportion of lobulation was higher than that of invasive adenocarcinoma; however, the number of cases of squamous lung cancer in this study was relatively small, and a larger amount of data was needed to confirm this.

A ground-glass nodule (GGN) is a confined, thin, hyperdense shadow observed on high-resolution CT. Depending on whether it contains a solid component or not, a GGN is classified as a mixed ground-glass nodule (mGGN) or a pure ground-glass nodule (pGGN). glass nodule (pGGN). Studies have suggested that thickening of the alveolar septa or fluid retention in their lumens, hemorrhage, and tissue debris are the pathologic basis for the formation of GGN (24). The changes in the content of solid components on HRCT images are manifested as changes in lesion density, which are achieved by tumor cell attachment growth, proliferation and infiltration during the formation process. Therefore, compared with solid nodules, mGGN has a higher malignancy rate and is closely related to the occurrence and development of lung adenocarcinoma (25, 26). This study showed that in lung adenocarcinoma, the ground glass sign was negatively correlated with the degree of pathologic malignancy, and the mixed ground glass sign and the proportion of solid were positively correlated with the degree of pathologic malignancy, and the nature of the nodule was of value in predicting the degree of malignancy, and the higher proportion of solid component of the lung nodule might suggest the higher degree of malignancy of the nodule.

Tumor markers are commonly used biological indicators for lung cancer detection and play an important role in tumor screening, diagnosis, efficacy observation and prognosis assessment (27). In this study, the differences in serum carcinoembryonic antigen (CEA) and squamous cell carcinoma antigen (SCCA) levels in patients with different pathological types of malignant lung nodules were statistically significant (*P* < 0.05), and the serum SCCA levels in patients with SC were significantly higher than those in patients with AIS, MIA, and IAC (*P* < 0.05), and the CEA levels in patients with IAC were significantly higher than those in patients with AIS and MIA (*P* < 0.05). CEA levels in IAC patients were significantly higher than those in AIS and MIA patients (*P* < 0.05). It is suggested that the combined detection of tumor markers may be important for the pathological staging of malignant lung nodules and the prediction of the degree of malignancy.

In summary, the clinical and imaging characteristics of patients with malignant lung nodules have a certain correlation with the pathological type, and gender, age, smoking history, nodule size, nodule nature, burr sign, pleural depression sign, and tumor markers are of great value for pathological typing. Especially, the correlation between imaging features and pathological types is stronger. Although there have been previous reports on the correlation between the degree of malignancy of pulmonary nodules and the clinical and imaging features of patients, this study focuses more on the differentiation of benign and malignant lesions. This study analyzes the correlation between malignant pathological types, imaging features, and clinical features, which has certain reference significance for more accurate determination of the nature of pulmonary nodules, treatment timing, and diagnosis and treatment methods. However, as this study is a single-center retrospective study, the inclusion of fewer cases may affect the reliability of the results, and a larger sample size is needed for validation. Combining the clinical and imaging characteristics of patients to determine the nature of lung nodules more accurately is of great significance for the early detection of malignant nodules, early diagnosis and treatment, improving the prognosis of patients, and reducing the waste of medical resources.

## Data Availability

The raw data supporting the conclusions of this article will be made available by the authors, without undue reservation.
